# Mechanical and Geometric Characterization of a Novel 2-Ply Vacuum-Pressed Biological Scaffold Patch Design for Posterior Mitral Valve Reconstruction

**DOI:** 10.1007/s12265-024-10572-0

**Published:** 2024-10-28

**Authors:** Johannes H. Jedrzejczyk, Frederik T. Andersen, Jacob Petersen, Alexander Emil Kaspersen, Urjosee Sahana, Søren N. Skov, Jens T. Væsel, J. Michael Hasenkam, Marcell J. Tjørnild

**Affiliations:** 1 Department of Cardiothoracic and Vascular Surgery, Aarhus University Hospital, Aarhus, Denmark; 2 Department of Clinical Medicine, Aarhus University, Aarhus, Denmark; 3 Department of Cardiothoracic Surgery, Odense University Hospital, Odense, Denmark

**Keywords:** Mitral valve reconstruction, Mitral valve repair, Posterior mitral valve leaflet, Vacuum-pressed, Lyophilized, Small intestinal submucosal extracellular matrix

## Abstract

To assess the mechanical properties of small intestinal submucosal extracellular matrix (SIS-ECM) iterations and choose the optimal version for evaluating functional geometrics after posterior mitral valve reconstruction. Four SIS-ECM versions (2- and 4-ply vacuum-pressed and lyophilized) underwent uniaxial tensile testing. A posterior mitral valve reconstruction patch was developed based on MRI scans (*n* = 5). Posterior mitral valve reconstruction using 2-ply vacuum-pressed SIS-ECM was performed (*n* = 7), and geometrics were evaluated using a modified left heart simulator. The vacuum-pressed iterations displayed superior maximum stress values compared to lyophilized (2-ply: median [IQR], 15.8 [15.2–19.0] vs 7.9 [7.3–8.3] MPa, p < 0.001; 4-ply: median (IQR), 15.8 –[14.6–22.0] vs 7.9 [7.6–8.4] MPa). All reconstructed valves were competent with preserved total leaflet area, but individual leaflet segment areas were redistributed. Posterior mitral valve reconstruction with our 2-ply vacuum-pressed SIS-ECM patch design was feasible in vitro. Further in vivo evaluation is warranted.

**Graphical Abstract:**

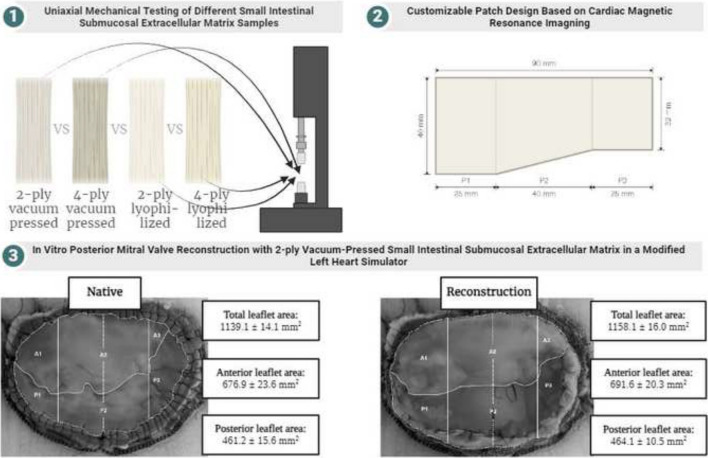

## Introduction

Mitral valve (MV) repair is typically preferred over replacement in surgical treatment for MV regurgitation or stenosis [1, 2]. Degenerative MV regurgitation caused by posterior leaflet prolapse was the first disease to be repaired [3]. However, repair may not be possible in severe posterior leaflet pathology cases due to insufficient pliable MV tissue or chordae tendineae caused by rheumatic valve disease, calcifications, infective endocarditis, or congenital heart defects [1, 4–6]. A patch repair can be done for minor defects in the MV leaflet, typically using a bovine or autologous pericardial patch. However, extensive damage to the posterior MV leaflet might warrant a replacement of the entire MV rather than repairing the posterior leaflet [1]. In such cases, a surgical solution that replaces the posterior leaflet and associated chordae tendineae while preserving the native anterior leaflet would be ideal for successful MV repair despite the damage to the posterior leaflet [7].

MV repair procedures using bio-scaffold materials are becoming more common [7, 8]. Small intestinal submucosal extracellular matrix (SIS-ECM), is a material that has been widely used in cardiovascular surgery [9]. We previously compared the biomechanical properties of the 2- and 4-ply lyophilized SIS-ECM material to native MV leaflet tissue [10]. The posterior MV leaflet tissue was found to be reminiscent of the 2-ply lyophilized SIS-ECM material, indicating that that 2-ply lyophilized SIS-ECM is a suitable option for repair of the posterior MV. Furthermore, we have proved it feasible to perform posterior MV repair using a 2-ply lyophilized version of the SIS-ECM material in vivo and in vitro [7, 8].

Recently, a new iteration of the bioscaffold was developed, using a vacuum-pressing technique to dehydrate the material instead of lyophilizing it. While the lyophilized versions of the SIS-ECM have a more porous microstructure, the vacuum-pressed versions have a more compact microstructure and are thinner than the lyophilized versions. Furthermore, the lyophilization process can cause changes in protein structure due to ice crystal formation, which does not occur when vacuum-pressing is used as the dehydration method [11].

While the 2- and 4-ply lyophilized SIS-ECM has been thoroughly evaluated, the vacuum-pressed version of the material has not yet been used for posterior MV repair. We hypothesize that the vacuum-pressed version of the SIS-ECM material has superior biomechanical properties in terms of stiffness, maximum stress and maximum load compared to the lyophilized version and is suitable for posterior mitral valve reconstruction in vitro. We aim to 1) compare the biomechanical properties of lyophilized and vacuum-pressed 2- and 4-ply SIS-ECM, 2) develop a personalized and customizable patch design for posterior mitral valve reconstruction based on magnetic resonance imaging (MRI), and 3) utilize the version of SIS-ECM with superior biomechanical properties for posterior mitral valve reconstruction using the proposed patch design and assess the geometrics in vitro using a modified left heart simulator.

## Methods

### Study Design

The biomechanical properties of four versions of the SIS-ECM material were evaluated through tensile testing to determine factors such as stiffness, maximum stress, and maximum load. The version of SIS-ECM with the most favorable biomechanical performance was chosen for in vitro posterior mitral valve reconstruction. A customized patch design for posterior mitral valve reconstruction was proposed based on geometric measurements from cardiac MRI scans of healthy pigs. Subsequently, in vitro posterior mitral valve reconstruction was conducted using a modified left heart simulator to compare the geometric parameters of the valve before and after the reconstruction.

### Uniaxial Tensile Testing of Small Intestinal Submucosal Extracellular Matrix

**Fig. 1:**
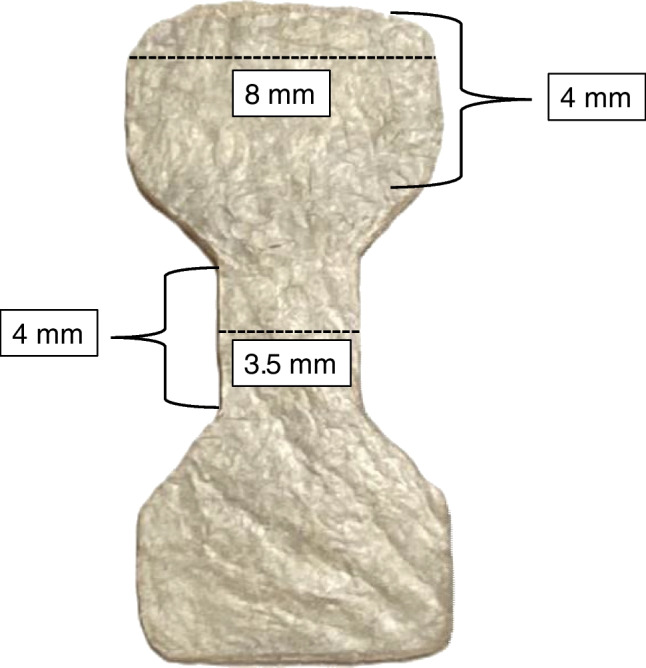
Dimensions of the SIS-ECM samples according to the American Society for Testing and Material tensile test standard

Four versions of the SIS-ECM material (CorMatrix®, Cardiovascular Inc., Alpharetta, GA, USA), including 2- and 4-ply lyophilized (0.14- and 0.28-mm thickness) and 2- and 4-ply vacuum-pressed (0.07- and 0.14-mm thickness) SIS-ECM, underwent mechanical uniaxial tensile testing. The SIS-ECM samples were cut into hourglass-shaped specimens using a dedicated die-cutter to ensure uniformity and reproducibility of the sample sizes. The hourglass shape is designed to prevent the samples from rupturing at the clamped areas, ensuring that any breakage occurs in the gauge area. After being cut, the dimensions of the gauge area were 4 × 3.5 mm, as shown in Fig. 1. The width and length of the samples were measured using calipers, while the thickness of the samples was measured using a thickness gauge with a constant pressure of 18 kPa (Model 7301, Mitutoyo Corporation, Kawasaki, Japan).

Before tensile testing, the SIS-ECM samples were hydrated in saline (Ringer-acetate, Fresenius Kabi, Fresenius SE & Co. KGaA, Bad Homburg vor der Höhe, Germany) for 10–15 min. The samples were tested in a Bose ElectroForce 3200 (Bose Corporation, ElectroForce Systems Group, Minnesota, USA) with a displacement sensor (+—0.001 mm) and a 225N load cell (+—0.002 N). Two custom gripping clamps were used for securing the samples during the experiments (G341-10–32 Needlenose Screw Vice Grip Rigid Mount, TestResources Inc., USA). The distance between the two clamps was incrementally increased at a rate of 0.5 mm/s and a sampling frequency of 10 Hz, leading to the eventual rupture of the samples. The samples underwent a 10 mm displacement, with displacement and force data recorded using a load cell in the tension machine.

### Definition of Stress–Strain Relationship

The samples were examined by a stress–strain relationship. Engineering strain ($\varepsilon )$ is defined as the change in length of a sample divided by its original gauge length [12]:

$$\varepsilon =\frac{L-{L}_{0}}{{L}_{0}}$$

Engineering stress ($\sigma$) is defined as the force divided per unit area [12]:

$$\sigma =\frac{F}{{A}_{0}}$$

where ${A}_{0}$ is the original cross-sectional area.

The stress–strain curve shows fundamental mechanical properties of a material. Initially, the samples exhibit elastic behavior with a linear stress–strain relationship, characterized by Young’s modulus ($E=\sigma /\varepsilon )$ [12].

Beyond the yield strength, permanent deformation occurs. The curve peaks at the ultimate tensile strength before the material weakens and ruptures, with fracture strain measured at the final data point.

### Posterior Mitral Valve Patch Design

**Fig. 2:**
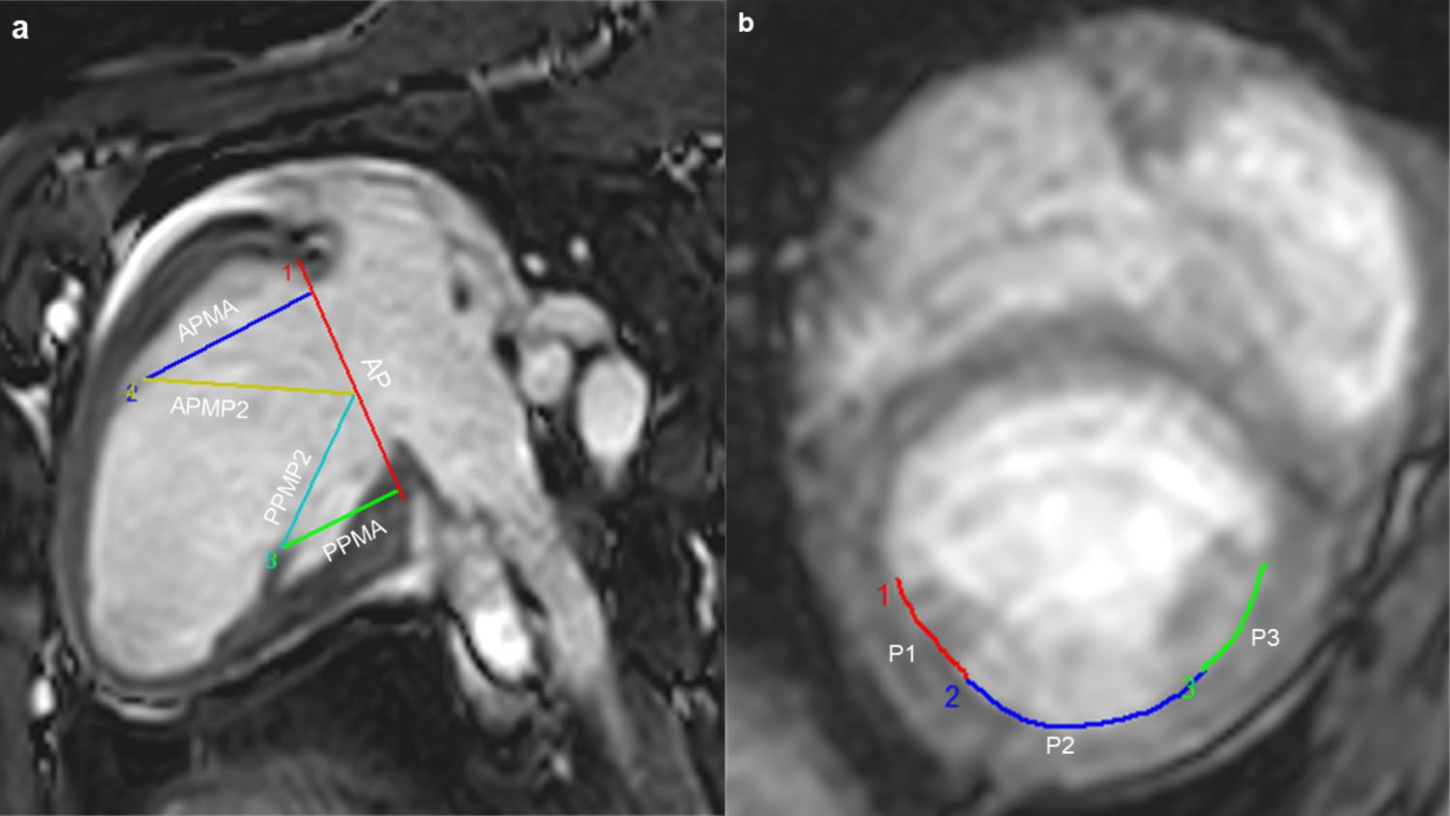
Geometrical measurements based on magnetic resonance imaging scans in **a** long-axis view of the heart in diastole and **b** short-axis view of the heart in diastole of five healthy, female 80-kg pigs used to propose the posterior mitral valve patch design. AP: annular plane; APMA: anterolateral papillary muscle to annulus length; APMP2: anterolateral papillary muscle to P2 length; P1, P2, and P3: posterior mitral valve scallops; PPMA: posteromedial papillary muscle to annulus length; PPMP2: posteromedial papillary muscle to P2 length

A 10 × 7 cm sheet of 2-ply vacuum-pressed SIS-ECM was used to create the posterior MV patch for reconstruction of the posterior MV leaflet and associated chordae tendineae. To accurately size the patch, we obtained MRI scans of five healthy six-month-old, 80 kg female pigs of mixed Yorkshire and Danish Landrace breeds from the University of Aarhus Experimental Animal Farm in Aarhus, Denmark. These scans were conducted to acquire geometric measurements of the mitral valve and sub-valvular apparatus (Fig. 2). During MRI, the animals were anesthetized by continuous intravenous administration of 4.375 mg/kg/h Propofol, and 6.2 µg/kg/h Fentanyl.

MRI scans were performed with a 1.5 Tesla Philips MRI scanner using a spine coil under the pig and an 18 elements anterior coil. The pigs were placed in a supine position. 2-dimensional cine images oriented around the MV were acquired in the short- and long-axis orientations. Furthermore, cine images oriented perpendicular to the MV were obtained (STACK1 and STACK2). For each stack, 8 slices with 6 mm thickness, a pixel size of 1.2 × 1.2 mm and 30 frames with a temporal solution of 32 ms were acquired. Finally, near-isotropic static 3-dimensional images at systole and diastole were acquired with a voxel size of 1.5 × 1.25 × 1.25 mm. The MRI data were stored, and the analysis was performed offline using software developed by MRI physicists at the Magnetic Resonance Research Centre, Aarhus University Hospital.

In a long-axis view, the distance from the fibrous head of the anterolateral papillary muscle to the middle segment of the P1 scallop and the distance from the posteromedial papillary muscle to the middle of the P3 scallop was measured. Similarly, the distances from each of the two papillary muscle tips to the middle of the P2 scallop was measured. In a short-axis view, the circumferential dimensions of each of the three posterior scallops (P1, P2, and P3) was measured. All distances were measured from inner edge to inner edge.

### Experimental In Vitro Setup

A porcine heart was chosen for this in vitro model, due to its similarity to the human heart physiology and anatomy [13]. The MV apparatus, encompassing the annulus, anterior and posterior leaflets, chordae tendineae, and anterior and posterior papillary muscles, was excised from the hearts of seven healthy six-month old, 80 kg female Danish Landrace pigs sourced from a local abattoir. Only MV apparatuses with intact chordae tendineae and matching dimensions of a Physio I™ annuloplasty ring size M36-M38 (Edwards Lifesciences, Irvine, CA) were included.

A power calculation based on a previous study with a similar setup was performed [8] using the equation below

$$n=2{\left(\left({z}_{\alpha }+{z}_{\beta }\right)\frac{\sigma }{\delta }\right)}^{2}$$

where z_α_ is the α-error of 0.05 and z_β_ is the probability of 85% (power) of which a change of 17% (δ) would be detected with an assumed standard deviation (σ) of no more than 8%. Using this formula, it can be found that *n* = 7.

### The Modified Left Heart Simulator

**Fig. 3:**
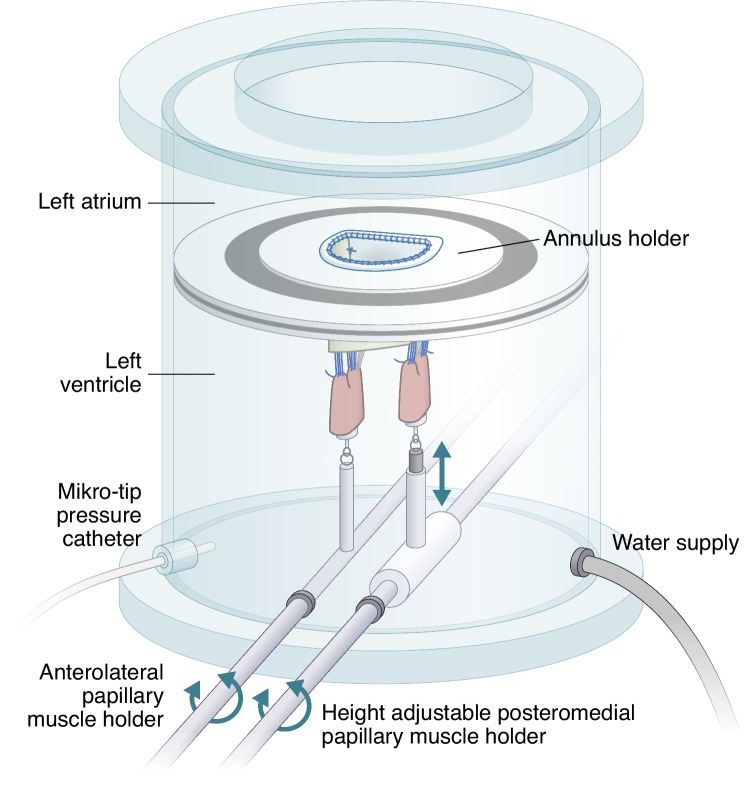
Schematic drawing of the in vitro left heart simulator

A modified left heart simulator, specifically designed for studying atrioventricular valves [8, 14, 15], was used for the functional geometric measurements. The model utilized a static flow and did not incorporate a pump or a compliance chamber. Tap water was used as the operating fluid. The simulator consists of an acrylic cylinder, an annular holding plate, and two papillary muscle rods (Fig. 3). The annulus holding plate, 3D printed to fit the annular proportions of a standard human MV, partitioned the acrylic cylinder into a ventricular and an atrial section. The inner circumference of the annulus holding plate was reinforced with Dacron® (Dupont, Wilmington, DE). The MV apparatus annulus was secured to the Dacron lining on the annulus holding plate with 5–0 Optilene® (B Braun, Melsungen, Germany) via a continuous interlocking suturing technique. Each papillary muscle was fitted with a fixture, secured with a 5–0 Optilene® suture. Subsequently, the fixture was securely fastened to its associated papillary muscle rod in the left heart simulator. For geometric measurements, a high-resolution camera was used to capture photographs of the MV apparatus from an atrial perspective.

A Mikro-Tip pressure catheter (SPR- 305S, Millar Instruments, Houston, TX) was utilized to measure left ventricular pressure in the left heart simulator. Since we have previously shown that a gradually increase in left ventricular pressure does not result in a significant geometric difference between the native and reconstructed MV, all geometric data were extracted at 120 mmHg [15]. Furthermore, maximum pressure tests were performed on all reconstructions to evaluate the short-term maximum resistance under static pressure. These tests involved gradually increasing the pressure until the valves ruptured. Finally, leaflet coaptation height was visually observed through the ventricular section chamber.

### Reconstruction of the Posterior Mitral Valve Leaflet

**Fig. 4:**
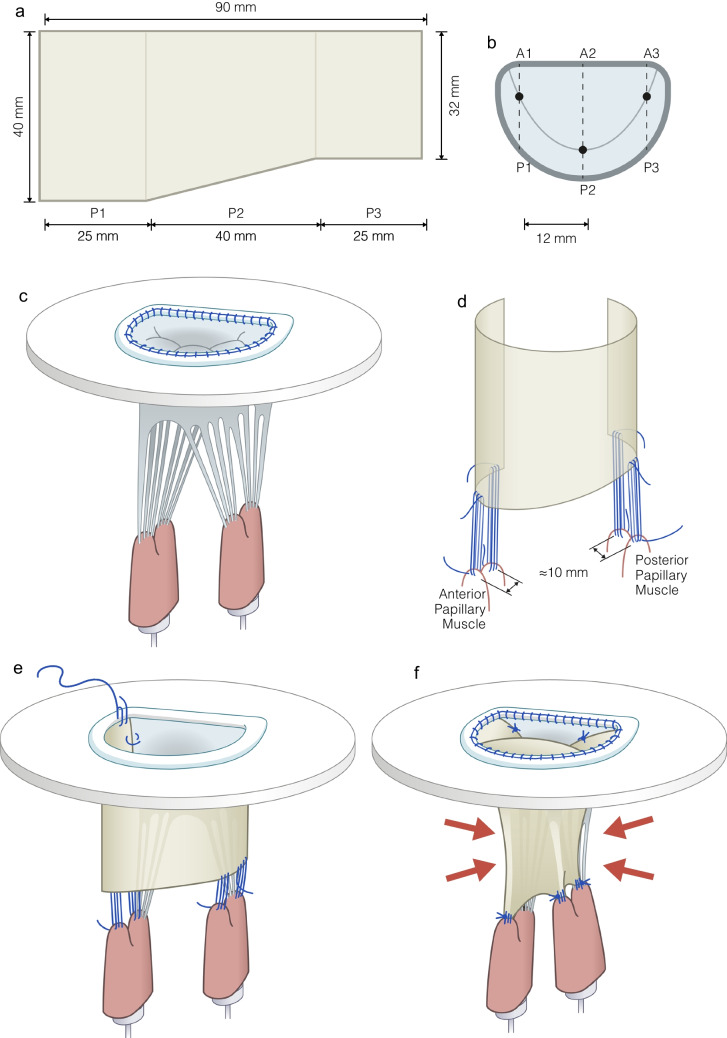
Schematic drawings of the posterior mitral valve patch design and implantation technique. **a** The specific dimensions of the patch design. **b** The measured A1, A2, A3 and P1, P2, P3 segments seen from a superior view. The big dots represent the coaptation point. **c** The native mitral valve implanted in the in vitro model. **d** The reconstruction, illustrating the 3-suture loop technique used to attach the four anchoring points of the patch to their respective papillary muscle heads, placed approximately 1 cm apart on each papillary muscle. **e** The patch being implanted in the in vitro model. **f** The patch implanted in the in vitro model. The red arrows indicate the applied ventricular pressure closing the reconstructed valve during systole

After collecting all the necessary data from the native MV apparatus, the posterior leaflet was surgically removed along with the corresponding chordae tendineae. Afterwards, the annular end of the leaflet patch was connected to the MV annulus with a 5–0 Optilene® continuous interlocking suture (Fig. 4). The patch was securely attached to the anterior leaflet at each commissure with a plication suture.

The ventricular extremity of the patch had four anchoring points. Both anchoring points were affixed to the apex of each papillary muscle, resulting in two connection points per papillary muscle spaced 1 cm apart. Three suture loops with 5–0 Optilene were employed to attach each anchoring point to the papillary muscle tips.

A crease was formed on the patch between the two attachment points on the individual papillary muscle. These two folds split the leaflet patch into three sections: P1, P2, and P3. The P1 and P3 segments served the purpose of replacing the P1 and P3 scallops as well and the chordae tendineae of the original posterior leaflet. This was achievable because the height of the leaflet patch was commensurate with the length between the native MV annulus and the tip of the native papillary muscle.

### Data Acquisition and Data Analysis

Utilizing the imaging software ImageJ (US National Institutes of Health, Bethesda, MD), the geometric data was extracted from all digital images manually. All images were assessed employing a 5 mm scale. This standard was established based on a shared aperture in every image. The software was utilized to identify the annulus, positioning it slightly within the sutures for both the native and reconstructed valves. This area was identified as the total leaflet area. The annulus and coaptation line demarcated the anterior and posterior leaflets. The A2 segment was delineated from the midpoint of the anterior annulus to the coaptation point and the P2 segment from the coaptation point to the posterior annulus. Combined, these two segments formed the A2P2 segment. Two lines delineated 12 mm apart from the A2P2 line, were designated as A1P1 and A3P3 and measured accordingly. A1, A3, P1, and P3 were gauged similarly. The same approach was used for both the native and the reconstructed valves, and the data was subsequently compared.

### Statistical Analysis

Normality of stress–strain and geometric data was assessed using histograms and quantile plots and tested using the Shapiro–Wilk test. Even though the stress–strain results of the lyophilized SIS-ECM was normally distributed, the stress–strain results of the vacuum-pressed SIS-ECM did not follow a Gaussian distribution and stress-stain data is expressed as median with interquartile range for easy comparison of the different SIS-ECM types. Comparison of 2- and 4-ply vacuum-pressed SIS-ECM was performed using Mann–Whitney U-test, while 2- and 4-ply lyophilized SIS-ECM was compared using independent t-test. The geometric parameters were normally distributed in the native and the reconstruction group, expressed as mean with standard deviation, and the two groups were analyzed correspondingly using dependent t-test. All tests were two-tailed and interpreted at a statistical significance level of 0.05. The statistical analyses were performed using SAS® Enterprise Guide® software, version 7.1 (SAS Institute Inc., Cary, NC).

## Results

### Uniaxial Testing of Small Intestinal Submucosal Extracellular Matrix

**Fig. 5:**
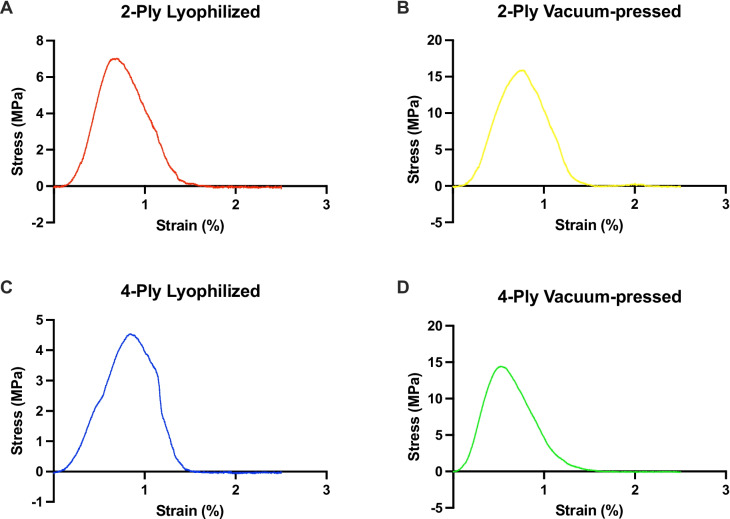
Stress vs strain graphs depicting the typical load response for each of the four different SIS-ECM samples. **a** Typical load response of the 2-ply lyophilized SIS-ECM. **b** Typical load response of the vacuum-pressed SIS-ECM. **c** Typical load response of the 4-ply lyophilized SIS-ECM. **d** Typical load response of the 4-ply vacuum-pressed SIS-ECM

Table 1 shows the biomechanical properties of the four different SIS-ECM versions. The typical load response for each of the four different SIS-ECM samples is depicted in Fig. 5. Comparing 2-ply to 4-ply vacuum-pressed SIS-ECM, a statistically significant difference in median stiffness (1.6 (1.4–2.0) vs 2.6 (2.2–4.4) N/mm, *p* < 0.001) and median maximum load (3.9 (3.7–4.7) vs 7.7 (7.1–10.08) N, *p* < 0.001) was observed. However, no statistically significant difference in median maximum stress was found (15.8 (15.2–19.0) vs 15.8 (14.6–22.0) MPa, *p* = 0.846). The comparison between 2-ply and 4-ply lyophilized SIS-ECM also revealed a notable difference in median stiffness (1.4 (1.3–1.5) vs 2.1 (1.8–2.3) N/mm, *p* < 0.001) and median maximum load (3.9 (3.6–4.1) vs 7.3 (6.9–7.7) N, *p* < 0.001). Additionally, no significant difference was observed in terms of median maximum stress (7.9 (7.3–8.3) vs 7.9 (7.6–8.4) MPa, *p* = 0.769). The comparison of 2-ply lyophilized, and 2-ply vacuum-pressed SIS-ECM revealed a statistically significant difference in median stiffness (1.4 (1.3–1.5) vs 1.6 (1.4–2.0) N/mm, *p* < 0.001) and median maximum stress (7.9 (7.3–8.3) vs 15.8 (15.2–19.0) MPa, *p* < 0.001). However, median maximum load (3.9 (3.6–4.1) vs 3.9 (3.7–4.7) N, p = 0.418) did not. Finally, when comparing 4-ply lyophilized and 4-ply vacuum-pressed SIS-ECM, a statistically significant difference was found in median stiffness (2.1 (1.8–2.3) vs 2.6 (2.2–4.4) N/mm, p < 0.001) and median maximum stress (7.9 (7.6–8.4) vs 15.8 (14.6–22.0) MPa, p < 0.001) but not in median maximum load (7.3 (6.9–7.7) vs 7.7 (7.1–10.8) N, *p* = 0.112).

**Table 1:** Biomechanical properties of the four different variations of small intestinal submucosal extracellular matrix

CorMatrix® SIS-ECM type	Stiffness (N/mm)	Maximum stress (MPa)	Maximum load (N)
2-ply vacuum-pressed	1.6 (1.4–2.0)	15.8 (15.2–19.0)	3.9 (3.7–4.7)
4-ply vacuum-pressed	2.6 (2.2–4.4)	15.8 (14.6–22.0)	7.7 (7.1–10.8)
2-ply lyophilized	1.4 (1.3–1.5)	7.9 (7.3–8.3)	3.9 (3.6–4.1)
4-ply lyophilized	2.1 (1.8–2.3)	7.9 (7.6–8.4)	7.3 (6.9–7.7)

Values are presented as median (interquartile range)

*SIS-ECM* small intestinal submucosal extracellular matrix

### Posterior Mitral Valve Patch Design

Based on the geometric measurements of the MV apparatus obtained by MRI (Table 2), a posterior MV patch measuring 90 mm in width, 40 mm in height at the P1 segment, and 32 mm in height at the P3 segment was proposed (Fig. 4a). The patch was visually divided into three segments: P1, P2, and P3 (25 mm, 40 mm, and 25 mm, respectively). The P1, P2, and P3 scallop folds were created when the patch was anchored to the papillary muscles. The patch was oversized at the papillary muscle attachment points, creating an upside-down isosceles trapezoid shape of P1 and P3, causing the P1 and P3 segments of the leaflet patch to constitute the chordae tendineae and the P1 and P3 scallops. The diagonal distance from the top of the P2 segment to the anchoring points at the base of the P1 and P3 (APMP2 and PPMP2) segments were oversized to allow free movement of the leaflet patch. Based on the MRI measurements, the circumference of the patch was oversized by 1.5 cm to allow for the reconstructed posterior MV leaflet to move forwards to the anterior leaflet in systole and secure a competent coaptation.

**Table 2:** Mean geometric measurements with standard deviation of the mitral valve based on magnetic resonance imaging scans of five healthy 80-kg pigs

Parameter	Diastolic phase (mm)	Systolic phase (mm)
APMA	33.1 ± 10.2	21.9 ± 6.4
APMP2	36.7 ± 8.2	26.9 ± 5.6
PPMP2	32.6 ± 6.5	23.5 ± 6.8
PPMA	26.3 ± 4.7	21.4 ± 5.5
P1	21.7 ± 3.5	15.1 ± 1.2
P2	37.4 ± 5.6	25.4 ± 3.9
P3	20.6 ± 0.7	17.8 ± 3.9
PLAC	79.7 ± 5.5	58.3 ± 5.8

Values are presented as mean ± standard deviation

*APMA* Anterolateral papillary muscle to annulus length, *APMP2* Anterolateral papillary muscle to P2 length, *PPMP2* Posteromedial papillary muscle to P2 length, *PPMA* posteromedial papillary muscle to annulus length, *PLAC* Posterior leaflet annular circumference

### Experimental In Vitro Setup

All valves were visually deemed fully competent during incremental increases in pressure from 0 to 120 mmHg. The posterior MV patch did not suffer any structural damage. Furthermore, the leaflet coaptation heights of the reconstructed and native MV were comparable. Nevertheless, an abundance of coaptation material was observed on the P2 of the leaflet patch compared with the anterior MV leaflet after reconstruction. The highest pressure achievable in all reconstructions was 260 mmHg before the onset of regurgitation. Upon an extensive review following the maximum pressure test, the sutures on the posteromedial papillary muscle had ruptured. There was no defect or tearing of the SIS-ECM material.

**Fig. 6:**
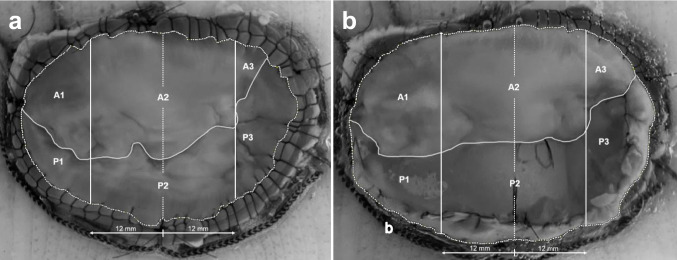
Representative images of closed valves seen from an atrial point of view comparing native **a** and reconstructed **b** mitral valves implanted in the in vitro model with annulus, coaptation lines and leaflet scallops

Table 3 presents the mean geometric measurements of the MV leaflets for native MV and after reconstruction with the posterior MV leaflet patch. Visual representations of the native valves and corresponding reconstructions with demarcated regions can be seen in Fig. 6. Neither total leaflet area nor individual leaflet area revealed any statistically significant differences. Nevertheless, the length of the A1 and A2 segments were statistically significantly reduced after reconstruction. Conversely, the length of the A3, P1 and P2 segments was statistically significantly increased after reconstruction. No statistically significant changes in length were observed for the P3 segment. Similarly, no statistically significant difference was found in the A1P1 segment when comparing the total leaflet segments. However, the A2P2 segment showed a statistically significant decrease in length after reconstruction, while the A3P3 segment exhibited a statistically significant increase in length following reconstruction.

**Table 3:** Mean in vitro geometric measurements with standard deviation of the native mitral valve and after posterior mitral valve reconstruction in seven porcine hearts

Parameter		Native valve	Reconstruction	*P*-value
Leaflet area (mm^2^)				
Total leaflet area		1139 ± 14	1158 ± 16	0.062
Anterior leaflet area		677 ± 24	692 ± 20	0.230
Posterior leaflet area		461 ± 16	464 ± 11	0.669
Leaflet scallops (mm)				
A1		20.5 ± 0.6	18.8 ± 0.5	0.003
A2		23.3 ± 1.1	18.5 ± 0.5	< 0.001
A3		14.6 ± 1.8	17.7 ± 0.6	0.008
P1		7.2 ± 0.4	8.5 ± 0.5	0.002
P2		9.0 ± 1.1	11.6 ± 0.4	0.001
P3		12.2 ± 1.6	11.8 ± 0.3	0.516
Total leaflet segments (mm)				
A1P1		27.6 ± 0.5	27.4 ± 0.8	0.631
A2P2		32.4 ± 0.2	30.3 ± 0.3	< 0.001
A3P3		26.8 ± 0.4	29.7 ± 0.6	< 0.001

Values are presented as mean ± standard deviation

## Discussion

We evaluated the biomechanical properties of four different versions of the SIS-ECM material. Based on the biomechanical results, we chose to use the 2-ply vacuum-pressed version to perform a complete in vitro surgical reconstruction of the posterior MV leaflet and associated chordae tendineae using our customized patch design based on MRI scans of healthy 80 kg pigs. The 2-ply vacuum-pressed SIS-ECM was chosen due to its unique mechanical properties and biocompatibility, making it ideal for mitral valve reconstruction. The 2-ply structure offers a thin yet strong structure, ideal for reconstructing the posterior mitral valve leaflet and providing a suitable foundation for regeneration. The vacuum-pressed SIS-ECM versions also showed the highest maximum load, maximum stress, and maximum stiffness, which is ideal for reconstructions in the mitral valve's high-pressure environment. Consequently, we chose the 2-ply vacuum-pressed version of the SIS-ECM for further investigations in the left heart simulator. All reconstructed valves were visually confirmed to function effectively with consistent leaflet areas pre- and post-reconstruction. Nevertheless, there was a noticeable redistribution of the individual leaflet segments (A1, A2, A3, P1, and P2) following the reconstruction.

### Uniaxial Tensile Testing of Small Intestinal Submucosal Extracellular Matrix

In our tensile tests, the vacuum-pressed SIS-ECM had greater stiffness and achieved higher maximum stress and load values than the lyophilized SIS-ECM. Lyophilization can cause changes in protein structure and material properties due to ice crystal formation [11]. Thus, we expected the vacuum-pressed versions to have higher biomechanical strength than the lyophilized versions. No results have been published on the biomechanical properties of vacuum-pressed SIS-ECM. However, our group has previously investigated the maximum load of lyophilized SIS-ECM. We found a median maximum load (95% CI) of 7.5 (7.0–7.9) N for 2-ply lyophilized SIS-ECM and 7.5 (7.1–8.1) N for 4-ply lyophilized SIS-ECM [10]. In the present study, we observed a discrepancy with our previous findings. Our results demonstrated that the 4-ply lyophilized SIS-ECM achieved a statistically significantly higher maximum load than the 2-ply lyophilized SIS-ECM despite using the same sample preparation and testing methodology. The observed differences might be attributed to changes in the manufacturing process. Minor biological variations in the small intestinal submucosa could impact the impact mechanical properties of the final SIS-ECM product. Additionally, parameters such as temperature, pressure and duration of the vacuum-pressing process influence the molecular structure and, consequently, the biomechanical behavior of the final product. Theoretically, slight deviations in these parameters could cause variations between different samples. It is also essential to consider the possibility of a batch effect. Even with quality control procedures in place, variations in production batches can arise from equipment calibration, operator handling, and environmental conditions during production.

We observed a considerable discrepancy in the maximum stress, i.e. mechanical strength, between the vacuum-pressed and the lyophilized versions of the SIS-ECM. Interestingly, the mechanical strength was very similar within the versions when comparing the 2-ply to the 4-ply configurations. The discrepancy in mechanical strength could be caused by the different processing methods used to create the materials. The lyophilization process is achieved by freezing the specimen and decreasing the pressure in the surrounding air, forcing water from the frozen phase to sublimate directly to the gaseous phase. This process can generate a porous structure with a higher surface area, which could result in reduced mechanical strength due to microvoids. The porous structure could benefit specific applications while potentially reducing mechanical strength. However, the reduced density and mechanical strength could decrease the durability and tensile strength of the material. Vacuum pressing involves placing the specimen in vacuum to eliminate air and consolidate the material. This method typically produces a denser and more homogeneous material with reduced voids. The enhanced density and decreased porosity increase mechanical strength and enhance structural integrity. The difference in mechanical strength between the lyophilized and vacuum-pressed samples can be attributed to fundamental variances in their microstructure and density. The results suggest that the preparation method substantially influences the mechanical strength, which previously has been proposed [11]. Lyophilized SIS-ECM is known to have a more porous structure with large amounts of separation artefacts compared with vacuum-pressed SIS-ECM, which has a denser and a more collapsed structure [11, 16]. These structural differences may explain the discrepancy in maximum stress when comparing the lyophilized and vacuum-pressed SIS-ECM versions. The 2-ply and 4-ply configurations of the vacuum-pressed and the lyophilized SIS-ECM showed similar mechanical strength, suggesting that the number of layers may not directly correlate to mechanical strength. However, the number of layers did have a direct correlation with stiffness and maximum load for both the vacuum-pressed and the lyophilized versions.

### Patch Design

The vacuum-pressed variations of the SIS-ECM are thinner and more robust than the lyophilized variants. Additionally, we hypothesize that the 2-ply version has a lower risk of delamination due to its reduced number of layers. However, this hypothesis must be evaluated in future research. Based on these findings and considerations, we selected the 2-ply vacuum-pressed version of the SIS-ECM material for our in vitro experiments for reconstruction of the posterior MV.

We have previously conducted a similar in vitro study with reconstruction of the posterior MV using a rectangular patch design with the 2-ply lyophilized version of the SIS-ECM [8]. This study utilized a novel, custom patch design to rebuild the posterior MV leaflets and its connecting chordae tendineae. In contrast to our former rectangular patch design, a “one size fits all”, the new design is intended to uniquely fit each recipient based on their anatomy to ensure the optimal outcome.

The patch was uniquely shaped, featuring three functional sections labelled P1, P2, and P3. P1 and P3 served as chordae tendineae substitutes, extending from the MV annular attachment to the papillary muscle attachment. The patch placement on each papillary muscle was only 1.0 mm apart on the anterior and posterior heads. At the MV annular level, the papillary muscles were spaced 25 mm apart, with their spatial orientation determined by the commissural and tertiary chordae tendineae on each side of P2. We used a three-loop suture technique to anchor the patch to the fibrotic tip of the papillary muscle heads. It is worth noting that the double folding of the patch material at the annular and papillary attachment, commonly applied in the lyophilized versions of the SIS-ECM material [17–19], has been omitted in our study due to the superior mechanical robustness of the vacuum-pressed version.

The reconstructed valves remained competent at the target pressure of 120 mmHg. A peak pressure of 260 mmHg was achieved before valve failure due to regurgitation. Interestingly, the SIS-ECM material remained intact without signs of tearing or rupture. However, upon examination, we found that the sutures at the annular attachment points had burst. As expected, we found no statistically significant change in total leaflet area, anterior leaflet area or posterior leaflet area before and after reconstruction at the target pressure of 120 mmHg. However, we observed statistically significant changes to the individual leaflet segments post-reconstruction. Although we ensured valve competence across all reconstructions, it remains unclear whether the redistribution of the individual leaflet segments will have clinical significance. Thus, further studies are required to evaluate our patch design in an 80-kg porcine in vivo model.

Including computational modelling within the design and analysis framework and the experimental methodology provides a way to perform a complete assessment. Predicting surgical outcomes and optimizing patch design can be achieved by using computational valve models to simulate the mitral valve biomechanics on a patient-specific basis. By describing the mitral valve in a manner that faithfully captures the complexity of the biomechanical environment, the effects of surgical intervention and design change can be fully explored and successfully implemented. Such models could be implemented into upcoming studies to accelerate the translation of this experimental approach towards a clinical application, thus providing a complete and predictive tool for surgical planning that instils confidence in the accuracy of predictions.

For future research, optimizations to the left heart simulator might allow for higher fidelity levels comparable to patient anatomical variations and improve the simulation of pathological conditions. Possible optimizations include features to replicate common mitral valve pathologies – such as leaflet prolapse, annular dilation, or calcification – and improving the operating fluid to resemble the properties of blood more closely. Glycerol may be used to adjust the fluid viscosity, while a saline solution may include suspended particles to replicate the cellular components of blood. With these optimizations, the left heart simulator could generate more accurate and robust assessments of mitral valve reconstructions under normal and simulated pathological conditions.

## Limitations

It is worth noting that the in vitro model used in our study has some limitations, as is typical with such setups. Thus, caution should be exercised when interpreting this data. First, the MV apparatuses used in the study were sourced from a local abattoir, which could have made them more fragile due to non-perfusion times. Second, the MV apparatuses included in the study did not have the pathologies typically associated with MV disease. Third, the left heart simulator used in the study simplified the complex interactions between the MV and subvalvular apparatus in a beating heart. It only simulated the systolic function of the MV within a limited range of fluid dynamic conditions compared to an in vivo setting. Fourth, our customized patch design was meticulously created for 80-kg pigs using MRI scans. Thus, our design might not be perfectly compatible with the annular holding plate used in this study. Lastly, the model used in the study had lower incremental increases in pressure and dLVP/dt (time derivative of the left ventricular pressure) compared to an in vivo setting.

## Conclusion

The vacuum-pressed SIS-ECM material displayed superior mechanical strength compared to the lyophilized versions. However, we did note a higher variability among the vacuum-pressed samples. Interestingly, the number of layers did not appear to impact the maximum stress of the material, though it did correlate with stiffness and maximum load for both vacuum-pressed and lyophilized versions.

Our new customized patch design for reconstructing the entire posterior MV apparatus achieved complete valve competence with the 2-ply vacuum-pressed SIS-ECM. The reconstructed valves remained functional up to a pressure of 260 mmHg, with no observed rupture or tearing. Notably, the total leaflet area, anterior leaflet area, and posterior leaflet area were preserved, though the area of the individual leaflet segments was redistributed after reconstruction.

This study suggests that the 2-ply vacuum-pressed SIS-ECM material shows potential for posterior mitral valve reconstruction. The combination of high maximum stress, stiffness, and load within a thin material, as observed in this study, could improve posterior mitral valve repair. However, to thoroughly evaluate the effectiveness of our surgical technique, further in vivo studies on an 80 kg porcine model, for which the patch was designed, will be necessary. If proven effective in vivo, this material could offer a valuable option for mitral valve reconstruction in the future.

## Data Availability

The datasets generated during and/or analysed during the current study are available from the corresponding author on reasonable request.

## Abbreviations

MRI: Magnetic resonance imaging
MV: Mitral valve
SIS-ECM: Small intestinal submucosal extracellular matrix
